# Exiting Surveillance From Abdominal Aortic Aneurysm Screening: The Views of Clinicians, and Men in Surveillance and Their Family Members

**DOI:** 10.1111/hex.70374

**Published:** 2025-08-28

**Authors:** Elizabeth Lumley, Jane Hughes, Alan Elstone, Jo Hall, Niall MacGregor‐Smith, Jonathan Michaels, Akhtar Nasim, Stephen Radley, Phil Shackley, Gerry Stansby, Emily Wood, Alicia O'Cathain

**Affiliations:** ^1^ SCHARR The University of Sheffield Sheffield UK; ^2^ University Hospitals Plymouth NHS Trust Plymouth UK; ^3^ Derbyshire Community Health Services NHS Foundation Trust Chesterfield UK; ^4^ PPI Member, c/o PCAAAS The University of Sheffield Sheffield UK; ^5^ Sheffield Vascular Institute Northern General Hospital Sheffield UK; ^6^ Newcastle Hospitals NHS Foundation Trust Freeman Hospital Newcastle upon Tyne UK

**Keywords:** abdominal aortic aneurysm, exit from screening, surveillance

## Abstract

**Background:**

The NHS Abdominal Aortic Aneurysm (AAA) Screening Programme in England screens men aged 65. Men with small aneurysms enter annual surveillance. The current ‘exit strategy’ is to leave surveillance after 15 years if the aneurysm remains small.

**Objectives:**

The aim was to explore the views of clinicians, men in surveillance and their family members about exiting surveillance.

**Design:**

A sequential study involving a Clinical Stakeholder Workshop to explore clinicians' views about factors that should be considered in any exit strategy, followed by a qualitative interview study to explore the views of men in surveillance and family members.

**Methods:**

A Clinical Stakeholder Workshop with 15 clinicians in the United Kingdom. Semi‐structured interviews with 22 men in surveillance and 5 of their family members from a single regional screening provider. Data were collected from January 2023 to April 2024. Framework Analysis was used.

**Results:**

Clinicians wanted an exit strategy to reduce unnecessary surveillance. They were concerned about the ethics of men attending for surveillance when they were not healthy enough for future treatment. They identified the need for a ‘low risk strategy’ for men with a low risk of future AAA rupture and a ‘poor health strategy’ so men could leave surveillance if they became too ill to attend surveillance or for future surgery. Men and their family members were less welcoming of an exit strategy because they valued the reassurance offered by surveillance. They also had an ethical concern about being removed from surveillance based on age. Some men proposed a reduction in the frequency of surveillance as an alternative to exit. Both clinicians and men valued shared decision‐making for exit from surveillance, whilst recognising that this needed to occur in the context of limited resources within the NHS screening programme.

**Conclusions:**

Although clinicians and patients had conflicting views about the need for an exit strategy from AAA surveillance, they agreed that shared decision‐making was key to any exit strategy.

**Patient or Public Contribution:**

This paper presents the perspectives of men with experience of abdominal aortic aneurysm (AAA) surveillance, and some of their family members. One member of the research team, who is also a co‐author on this paper, is a man who was diagnosed with an AAA. He actively contributed to the design and delivery of the study as co‐applicant on the funding grant. He also attended monthly project meetings where decisions were made about how best to conduct the research. We set up a new Patient and Public Involvement (PPI) Panel made up of men who had experienced AAA screening, including four who were diagnosed with AAA. This panel met eight times throughout the project to ensure that the interview invitations and the topic guide were appropriate; to interpret the findings; and to advise on dissemination strategies.

## 1 Introduction

### 1.1 Abdominal Aortic Aneurysm

An abdominal aortic aneurysm (AAA) is a swelling or bulge in the aorta that is larger than 3 cm. AAA can increase in size over time and may rupture [1]. As the aorta is the main artery that carries blood from the heart to the abdomen, this causes internal bleeding that can be fatal [1]. In men, rupture is uncommon until the AAA reaches 5.5 cm or greater [2]. 3000 people die each year in England and Wales from a ruptured AAA [1]. Screening to detect AAA is important as the majority of those with an AAA have no symptoms, and early detection via screening has been shown to reduce population mortality rates from ruptured AAA [3].

### 1.2 AAA Screening and Surveillance

Screening programmes differ between countries. National AAA screening programmes have been introduced in a number of countries, including Sweden, England, Wales, Scotland and Northern Ireland [4, 5]. In the United States, AAA screening is recommended for men aged 65‐75 years who have ever smoked [4]. In Italy, Western Australia and New Zealand, regional screening programmes have been trialled [4, 5]. Screening programmes tend to focus only on men. Only the United States, New Zealand and Italy include women in their screening programmes [4].

There is a National Health Service (NHS) in England that provides care free at the point of access. The NHS AAA Screening Programme in England offers ultrasound screening to all men in the year they turn 65. Only men are screened because they are six times more likely to have an AAA than women [3]. The NHS AAA Screening Programme started in a small number of regions in 2009, becoming fully national in 2013 [6]. Men diagnosed with AAA enter surveillance. Those with a AAA classified as ‘small’ (size 3–4.4 cm) have annual ultrasound scans, whilst those with a AAA classified as ‘medium’ (size 4.5–5.4 cm) have 3‐monthly scans. Between April 2021 and March 2022, approximately 14,600 men in England were under surveillance [7].

### 1.3 Exiting Surveillance

When the NHS AAA Screening Programme in England was established, it included guidance that men would exit surveillance if, after 15 scans at 1‐year intervals, the AAA remains small (below 4.5 cm) [8]. This would apply to men who had been in annual surveillance for 15 years, usually aged 80 at the point of exit. This guidance is now relevant to a small number of men who joined surveillance offered by a few regional screening providers in 2009. It will become relevant to thousands of men in 2028 as the national programme reaches 15 years in operation.

The current exit strategy is focused on the low risk of rupture. By implication, the age of the affected men is 80 years. Whilst in surveillance, as men age, they may develop health conditions that can mean they are unfit for surgery if they develop a large AAA. Some men experience ‘Turn Down’ where they are referred for surgical repair but are not healthy enough to have this intervention [9]. This group is small but will continue to grow each year, leading to concerns that this could threaten the value of the programme [9]. The issue of how best to deal with men who enter or remain in surveillance when they are likely to be unfit for surgery is a topic of debate amongst vascular surgeons [10, 11, 12].

It is timely to explore the acceptability and the comprehensiveness of the exit strategy from AAA surveillance in England for a number of reasons. First, it may be a significant waste of resources to continue AAA surveillance for men who will never require surgery; therefore, an appropriate exit strategy has potential economic benefits for a healthcare system. Second, the ethical appropriateness of the current exit strategy has not been explored.

## 2 Methods

### 2.1 Aim

The aim was to explore the views of clinicians, men in surveillance and their family members about exiting AAA surveillance.

### 2.2 Design

This was a sequential study. First, we undertook a Clinical Stakeholder Workshop to identify clinicians' views about the factors that should be considered in any exit strategy. Then we undertook a qualitative interview study of men in surveillance and family members of men in surveillance to explore their views. This was undertaken as part of wider study called the Patient‐Centred AAA study (PCAAAS) (https://www.sheffield.ac.uk/scharr/research/centres/hcru/pcaaas).

### 2.3 Ethics

Approval for the Clinical Stakeholder Workshop was received from The University of Sheffield Research Ethics Committee—application number 050427. Approval for the qualitative interviews with men in surveillance and their family members was granted by the WalesRec6 Ethics Committee 23/WA/0019, IRAS project ID 321528.

### 2.4 Clinical Stakeholder Workshop

The NHS AAA Screening Programme in England is delivered by 38 regional providers. We aimed to invite clinicians linked with a variety of these providers. We sought to include vascular surgeons who provide surgical repair for large AAAs (*n* = 4); clinical directors/leads of regional screening providers who oversee the programmes within their region, including effectiveness, quality and safety (*n* = 4); and AAA screening specialist nurses (*n* = 4) who provide health and lifestyle advice, support and reassurance, and manage surveillance follow‐up. We invited these clinicians using a list of names and addresses of regional providers. We also invited anaesthetists because they make decisions about whether patients are fit for surgery.

Our research team consisted of clinicians and methodologists, including three vascular surgeons (one of whom was the Clinical Director of the NHS AAA Screening programme), a clinical director of a screening provider, and an AAA screening specialist nurse. All these clinicians attended and participated in the workshop, bringing expertise in AAA screening. A fourth vascular surgeon approached us to attend because he was interested in exit strategies outside of the NHS screening programme. If clinical stakeholders agreed in principle, a member of the research team sent them a letter of invitation, a participant information sheet and a consent form. We paid the employing organisations for 3 h of their staff time using staff hourly payment rates.

We held a 2‐h virtual workshop in January 2023. The research team presented national and international evidence and published debates about exit strategies from AAA surveillance [10, 11]. Factors for discussion included: age of men, size of AAA, rate of growth of AAA, health status/fitness, risk of surgery versus risk of AAA rupture. The meeting also included discussion of how and when decisions should be made and communicated. The meeting was recorded, and field notes were taken by two research team members. We transcribed the workshop discussion verbatim, and comments placed in the ‘chat’ section of the virtual meeting software were included in the transcript.

### 2.5 Qualitative Interview Study With Men and Family Members

We undertook qualitative interviews with men in the surveillance programme and their family members by recruiting from a single regional screening provider based in the North of England. We undertook maximum diversity sampling to include men of different ages, with different health profiles, who had different sizes of AAA, different rates of growth and different lengths of time in surveillance. We had two routes of recruitment for men. First, the regional screening provider gave us a list of names and addresses of men in the AAA surveillance programme who had consented to be contacted about research. We sent an information pack including an invitation letter, a participant information leaflet, a consent form and a response card with a freepost envelope. Men were asked to contact the research team if they were interested in taking part in an interview. Second, in the wider study, we undertook a survey of men in surveillance, most of whom agreed to be involved in other parts of the study. We wrote to some of these men, inviting them to participate in this part of the study. For family member recruitment, we asked men during each interview if any family members were aware of their AAA and involved in providing any kind of support or attending screening with them. Those who indicated that they did have this input from family were asked to approach their family member about an interview. The man then passed the contact details of the family member to the research team if they consented to this. We also tried to recruit family members of men in AAA surveillance using social media, without success.

Interviews were undertaken by telephone or a virtual platform and lasted between 20 and 46 min (average 32 min). The topic guide for the interviews was developed based on topics that arose from the Clinical Stakeholder Workshop and included questions about personal experiences of being in surveillance, opinions about the current exit strategy, and how decisions should be made. Interviews were audio recorded and transcribed verbatim.

### 2.6 Analysis

One researcher (A.O.C.) undertook framework analysis of the transcript and field notes for the clinical stakeholder workshop [13]. One researcher (E.L.) undertook framework analysis for the qualitative interviews. The framework was selected because there were specific research questions, such as what factors should be considered in exiting surveillance and what process should be used when considering exit. Some factors had been identified in the literature [10, 11]. The specific research questions and factors offered a framework for coding the data, with additional attention to themes identified inductively. Framework analysis involved reading the transcripts to become familiar with the data, constructing a framework of themes and sub‐themes based on the research questions and reading the transcripts, coding all transcripts using the framework, and then considering the links between the themes and sub‐themes. Preliminary findings were discussed with the wider team and the Patient and Public Involvement (PPI) Panel (see earlier for a description of this panel). The PPI group was supportive of the findings from the men's interviews and felt that they reflected some of the concerns they had themselves about the exit strategy. Then A.O.C. and E.L. used a triangulation protocol to put the findings from the two data sources side by side, comparing the clinician and men/family members' views [14].

The findings presented are largely descriptive because it was important to report the views of different stakeholders about different aspects of an exit strategy. There is some interpretive analysis regarding the ethics of exiting surveillance. Quotes are used to illustrate the findings and labelled using the interview number (e.g., ES1 for Exit Strategy participant 1 and SES1 for family member associated with ES participant 1) and the size and growth rate of AAA.

## 3 Results

### 3.1 Participants

#### 3.1.1 Clinical Stakeholder Workshop

23 clinicians were invited to the meeting. Seven did not respond to the invite, and three were unable to attend on the date specified. 13 clinicians confirmed attendance at the meeting. On the day, two did not join, leaving 11 external clinician attendees and 4 clinicians from the research team (15 clinicians in total). The spread was good across different clinical roles (see Table 1).

**Table 1:** Participants at the clinical stakeholder workshop.

Role	External clinical attendees	Research team clinical attendees	Total
Consultant Vascular Surgeon	1	2	3
Consultant Vascular Surgeon and Screening Clinical Director	3	1	4
Vascular Nurse Specialist or Vascular Nurse	3	1	4
Nurse Specialist and Programme Manager	1		1
Consultant Anaesthetist	2		2
Quality and Assurance Manager and Programme Manager	1		1

#### 3.1.2 Qualitative Interview Study

154 men were invited to an interview. 28 men responded, and 22 men took part in an interview. Of the six that did not take part, five did not respond to attempts to arrange an interview date and one did not attend the agreed‐upon interview. Five family members of men in surveillance were interviewed (four wives and one daughter). We had aimed to interview 10–12 family members, but they tended to decline the invitation because they reported little knowledge of the man's AAA experience. Social media recruitment yielded no response from family members.

We collected demographic information on the men (see Table 2). We had a wide range of ages, social deprivation levels and AAA growth rates. No men were from ethnic minority groups. Men mainly had small AAAs because the existing exit strategy focuses on them.

**Table 2:** Characteristics of men interviewed.

Characteristic	Number of participants
Age range (mean)	66–82 years (mean 69 years)
Ethnicity	
White	22
Ethic minority group	0
Indices of Multiple Deprivation quintiles	
1 = most deprived	7
2	5
3	2
4	5
5 = least deprived/most affluent	3
Reported size of AAA	
Small	14
Medium	6
Large	2
Perceived growth rate	
Non‐growing	9
Growing Slowly	9
Growing Quickly	4
Scan frequency^a^	
Annual	15
3‐monthly	5
6‐monthly	2
Time in surveillance	
1–2 years	3
3–4 years	1
5–6 years	10
7–8 years	0
9+ years	8

^a^ One man interviewed reported having a medium AAA but having annual screening. We had no means of checking its accuracy.

### 3.2 Overview of Themes

We present three themes. The first theme ‘Exiting surveillance—a welcome prospect?’ explores the differing reactions of clinicians and men/family members, where clinicians welcome an exit strategy and men question the need for one. The ethical issues surrounding exiting surveillance partly explain these differing views. The second theme ‘More than one type of exit strategy’ describes alternatives to the current exit strategy. The third theme ‘Making decisions about exiting surveillance’ describes views about preparing men for exit, when to make the decision and with whom, and the importance of shared decision‐making around exiting surveillance.

### 3.3 Exiting Surveillance—A Welcome Prospect?

While clinicians were generally supportive of having an exit strategy, men and family members found reassurance in having regular scans so they could monitor the growth rate of their AAA.

#### 3.3.1 A Necessary Endeavour

The clinicians were generally supportive of having a guideline on exiting surveillance because this helped to maintain the cost‐effectiveness of the screening programme and addressed an ethical dilemma of keeping men in surveillance who would be too ill to have treatment if their AAA became large enough for repair.

‘they may have significant COPD, significant ischemic heart disease, they may have cancer and the question is, if they continue for 10‐15 years and […] referred for intervention, and you say “you're not fit” […] and the man turns around and says “well, I've been attending surveillance for 10 years, why didn't somebody tell me that it's all been unnecessary? Why have I been through all this and all the anxiety”’

Vascular surgeon 1

The clinicians varied in view from enthusiasm for immediate action, particularly for men too ill to have surgery if their AAA reached a certain size, through to having concerns about another ethical issue that would need to be considered before introducing an exit strategy (see later). Some of the screening programme directors and programme managers were also driven by their programme's performance, expressing concern that if some men exited by simply not attending surveillance, then this would affect a service's key performance indicators. They felt that a formal procedure for exiting would allow for the removal from performance statistics of men who preferred not to attend. Clinicians felt there was an evidence gap around men's preferences around exit from surveillance, so they welcomed the planned interviews with men.

#### 3.3.2 Surveillance Offers Reassurance

The men and family members expressed surprise at the existence of an exit strategy, believing that surveillance would continue until the AAA reached the size for referral for surgery or the man died. Although some men supported the need for an exit strategy because it would only apply to those with small aneurysms who therefore had a low risk of future rupture, other men and their family members' preferences were in sharp contrast with clinicians' views. They did not want to lose the reassurance and peace of mind they obtained from surveillance. All family members interviewed were against the idea of an exit strategy, mainly because they perceived it as too risky to end surveillance. This issue of risk was highlighted by a man who expressed concern that the risk of rupture remained, even if it was low risk, citing his experience of losing a relative to cancer reoccurrence after he had exited years of post‐cancer surveillance. Views did not seem to depend on men's size of aneurysm or rate of growth.

‘Well like I said before you know if the growth is so small, is it worth keeping an eye on it you know?’

ES20 (medium slow‐growing AAA)

‘Well, to be perfectly honest I would rather have it to reassure every year, even after 15 years just to be on the safe side. That is assuming I live that long.’

ES23 (small non‐growing AAA)

#### 3.3.3 The Ethics of Exit

As described earlier, some clinicians wanted an exit strategy to prevent an ethical problem of offering surveillance to people who would not receive treatment in the future. Other clinicians raised a different ethical concern, that surgical intervention might improve over time and be safer for men in poor health in the future. Some men also raised this latter concern. Men who did not find an exit strategy acceptable raised the ethical concern that an exit strategy might prioritise saving money over patients' health. These men and family members viewed any consideration of an exit strategy as a cost‐saving endeavour, and some discussed how they would pay for scans privately if they were exited from surveillance.

‘Well, if I'm honest not very comfortably because, well it gets me thinking about why has there been a surveillance for a number of years if at a certain point in time you're just going to be taken off the scheme. It's nearly as if, well, we might have done something about it if it had happened while you were from 60 to 80, but after that, it's a bit of a message of, well, it's not really that important, so, that doesn't sit very comfortably with me.’

SES2 (Family member of man with a small slow‐growing AAA)

### 3.4 More Than One Type of Exit Strategy: Low Risk Versus Poor Health Versus Reduced Surveillance

During the Clinical Stakeholder Workshop, clinicians described two types of exit strategy that we characterise as ‘low risk’ and ‘poor health’. A ‘low risk’ strategy focused on exiting men with a low risk of progression to a large AAA and potential rupture. A ‘poor health’ strategy focused on excluding men from surveillance who were already too ill to have repair surgery and would likely be turned down for surgery if referred, or who struggled to attend surveillance due to poor health. Clinicians did not consider these two types of strategies as alternatives, but rather as complementary; that is, raising the option of having multiple parts to an exit strategy.

During the qualitative interviews we introduced men and family members to the existing strategy (which is a ‘low risk’ strategy based on size of AAA after 15 years, with an implicit ‘poor health’ strategy in that men aged over 80 might be too ill for treatment) and the two types of strategies identified by clinicians (low risk and poor health).

#### 3.4.1 ‘Low Risk’ Strategy

Some men in the sample supported the rationale of exiting based on the low risk of rupture because it was safe. Indeed, some men viewed the age of 75 (10 years of surveillance) as more appropriate for exit for men with small aneurysms, as they did not see a big difference between 10 and 15 years in surveillance. However, one of the men pointed out that the risk of rupture did not necessarily remain stable, and the risk could increase post‐exit.

‘…if it's going to be growing then keep scanning it, if it isn't, to my mind, there's no point. If you have two or three scans and it's exactly the same each time you go, is there much point in going?’

ES15 (large quick‐growing AAA)

‘so although they may have been stable for 15 years I guess the critical period might be in the next five or ten.’

ES3 (small slow‐growing AAA)

#### 3.4.2 ‘Poor Health’ Strategy

Clinicians described how some men attending for screening were very ill, affecting their capacity to make decisions (e.g., severe dementia), making attendance for screening burdensome (e.g., on home oxygen) or likely to be turned down for surgery if referred (e.g. had comorbidities). Some clinicians proposed that the existing exit strategy could be adapted so that men over 85 or 90 with small aneurysms would leave surveillance, using age as a proxy for poor health. They wanted to raise the number of years in surveillance (and therefore the age of the man) in recognition of the number of healthy men in their 80s who might benefit from treatment if their AAA became large. Other clinicians preferred that age not be used as a proxy for poor health because they felt that men of the same age could have varying levels of health status. They proposed that a better characteristic for exiting surveillance was frailty, while recognising that this was not straightforward to assess.

‘I suppose I just want to reinforce that you've got potentially a group of people who are not fit for any intervention, either because of their comorbidities or because of the anatomy of their aneurysm. So that's a very high risk of turn down, no matter what the screening shows’

Anaesthetist 1

For the men interviewed, some felt that 80 was too early for any exit strategy to be implemented, and that they felt like they were being ‘written off’ when they were still in good health; this was echoed by the family members interviewed. Like some of the clinicians, they proposed 85 (i.e., 20 years of surveillance) as a more appropriate age, or the use of individual health status rather than age for decision‐making about exit.

#### 3.4.3 A New Strategy: ‘Less Frequent Screening’ or ‘Opt Back In’ Strategies

Alternative exit strategies were proposed by men: dropping the surveillance frequency to 2‐yearly or 5‐yearly scans rather than leaving altogether; or, as with bowel and breast screening programmes, having an option to self‐refer back into surveillance if they had any concerns.

‘I would have thought maybe after 5 years at the same reading and it's low then you could go every 2 years maybe or every 3 years even rather than going every year for another ten years’

ES1 (small non‐growing AAA)

‘my view is that instead of being annually, make it 5 yearly, but you've got to also give the option that if somebody says “hang on I'm not feeling so well here and I'm wondering if it's this AAA that's causing me problems, then can I have another scan.” Then you get the best of both worlds don't you at the end of the day.’

ES15 (large quick‐growing AAA)

### 3.5 Making Decisions About Exiting Surveillance

Clinicians and men tended to hold differing views about the decision‐making process. In terms of preparing for the decision, clinicians focused on men's expectations as a way of improving decision‐making about surveillance, while men exhibited knowledge gaps about AAA and screening that would hinder any decision‐making. For timing of the decision, clinicians and some men favoured early discussions about exit, while other men expressed ethical concerns that this might be unnecessary and cause anxiety. The preferred clinical decision‐maker was shaped by resource issues for clinicians, while men were interested in the knowledge levels of the clinical participants. Both groups agreed that shared decision‐making was the favoured way forward.

#### 3.5.1 Attending to Men's Expectations and Knowledge to Help Them to Engage in Decision‐Making

Some clinicians felt that the surveillance programme led men to believe that they *should* attend surveillance, and that it could be clearer that they have a choice to leave if they wish. They also highlighted that there might be a need to change men's expectations that they would have a repair if their aneurysm reached the treatment threshold size. They felt that Specialist Nurses and Technicians could be trained to ensure that surgery was discussed as a potential rather than a certain outcome. Related to this, they considered there was a need to educate men from the beginning of surveillance about how they might become too ill for repair. This could also be an opportunity to discuss health optimisation, encouraging some men to become fitter to increase the chance of having surgery if needed.

‘They have an expectation of it's leading to something. So if you did put in place an exit strategy, I think that would need to be discussed with them from the beginning as opposed to us just telling them that they're entering surveillance that we're going to keep monitoring it’.

Programme Manager 1

During the interviews with men and their family members, it became clear that, as well as not knowing about the existing exit strategy, they had a lot of knowledge gaps about AAA and AAA screening. One man described not having heard the word ‘aneurysm’ before. Men described not understanding what might cause an AAA, what might cause it to grow and what any concerning signs and symptoms were that they should look out for. These gaps in understanding were reported by men and family members across all AAA sizes, growth rates, lengths of time in surveillance, and socio‐economic groups. This highlighted an ethical challenge of having meaningful discussions about leaving surveillance if men and their families lacked understanding about AAA and surveillance.

#### 3.5.2 When to Have the Discussion About Exiting Surveillance

Clinicians proposed that the right time for discussions about exiting surveillance was during the Specialist Nurse appointment when men first entered surveillance. This discussion could focus on setting the right expectations around surveillance and treatment, educating men about AAA, engaging with men's concerns, and discussing the potential for later exit from surveillance. Nurses in the Clinical Stakeholder Workshop felt that some of this information had already been given, but acknowledged that there was limited time for detailed discussion during these appointments. Clinicians also raised the possibility of discussing exit whenever men attended for scans, or when they reached a certain age or length of time in surveillance. They raised the possibility of offering another review discussion with a Specialist Nurse in addition to the two already in place (on entering surveillance and one on moving from annual to 3‐monthly surveillance).

Men and their family members expressed two very different views. Some men believed discussions about exit should start early in the pathway, so that they had all the facts from the beginning and there would be no surprises down the line. They also felt that the discussions would need to be repeated over the years of surveillance. In contrast, the others preferred to wait until they had been under surveillance for a few years, because they thought that, if it was done too early, it might result in an overwhelming amount of information to take in, be frightening, or may not be relevant to some men if their AAA grew over time. Two men proposed an individualised approach, with discussions about whether men had other health conditions that might reduce the value of surveillance. The size and growth rate of AAA seemed to have no bearing on views about the timing of any discussion.

‘I would say definitely the first time they're diagnosed and treatment, but I would say maybe every what 2 or 3 years.’

ES23 (small non‐growing AAA)

‘I suspect for some people that might be a bit too much to take in and it might be a waste of everybody's time too early in the process because there might be a tendency to—“no that's in the future I'm worried about the here and now” and they push that to one side.’

ES26 (small slow‐growing AAA)

#### 3.5.3 Who to Discuss Exit With

Concerns about the limited resources available to the NHS screening programme affected clinicians' views about any discussion with men about exiting surveillance. They discussed the ‘low resource’ option of sending a questionnaire to men when they get to a certain age, with information about the pros and cons of surveillance, and asking them if they wanted to exit. They also discussed a ‘high resource’ option of doing physical assessments and tests on men to assess frailty, but dismissed this as too expensive to implement. If a discussion about exit were to take place, they did not think that Screening Technicians could be trained to have these conversations, but did suggest that they could have a checklist to raise a red flag (frailty or comorbidity) and refer men to Nurse Specialists for a discussion. They identified Specialist Nurse appointments as appropriate opportunities for a discussion, but suggested that men with identified comorbidities that might make them unsuitable for surgery could be referred to a vascular surgeon or the clinical director of the screening provider for a discussion about the risks of surgery in the future. This would result in only a small number of men seeing a surgeon.

‘where the nurse meets them for the first time or when they change from medium to large, if they could, the nurse could escalate it to the consultant to see them and stop the unnecessary surveillance’.

Vascular Surgeon 2

Men were more concerned about talking to the right person. They were happy to talk to anyone in the screening service as long as the person had the right information, could answer any questions raised, had good communication skills, and could refer them to a specialist doctor or nurse if necessary. They did not consider GPs to have the accessibility or knowledge required. Some men expressed a strong preference to talk to a surgeon about fitness for surgery.

#### 3.5.4 The Need for Shared Decision‐Making

The clinicians recognised the importance of shared decision‐making around exit and the need to listen to men's concerns. They recognised that men might want to stay in surveillance even if they were at low risk of rupture or not fit for surgery, and that men would need information to help them decide.

‘the patients being aware of risk versus benefits. So, maybe a bit more information for the patients to make that decision as to whether they want to stay on […] it's probably more information for the patients’.

Specialist Nurse 1

Men and family members were in agreement with clinicians that they should have a say in whether or not they should exit surveillance. Only one man (small non‐growing AAA) disagreed, but he recognised that most men probably would want to be involved in decision‐making. Men acknowledged that it might not be an easy decision to make, and that it would be important to have the right information. A few men reported that they would want to discuss the decision with family or friends, whilst not necessarily wanting them to influence or change their decision.

## 4 Discussion

Clinicians wanted an exit strategy to stop unnecessary surveillance. They were concerned about the cost‐effectiveness of the screening programme and the ethical problem of keeping men under surveillance when they were not healthy enough to attend for scans or have treatment in the future. Men and their family members were less welcoming of an exit strategy because they valued the reassurance offered by surveillance. They also had ethical concerns, but these were about the current exit, which they believed did not value the health of people over 80. Some men favoured a reduction in the frequency of scanning as an alternative to exiting surveillance. Both clinicians and men valued shared decision‐making for exit from surveillance while recognising that this needed to occur in the context of limited resources within the NHS.

Our findings were similar to a recent study undertaken in another part of the United Kingdom—Wales—outside the context of the NHS screening programme. Twenty‐four men and women aged over 85 years with small AAA were interviewed about exiting surveillance and their preferences measured [15]. When offered an exit from surveillance, two‐thirds decided to stay in surveillance because they wanted the reassurance it offered, and a third chose to exit. The study also found poor levels of knowledge about AAA, for example, believing that rupture was imminent when the AAA became large. Another study examined the impact of a consultation with a vascular consultant with participants over the age of 85. It found that 40% of 42 people chose to exit surveillance following a short (median 10 min) consultation [16]; it is not clear what was discussed in the consultation or whether a patient decision aid was used.

Some of our interview participants suggested that, rather than leaving surveillance altogether, the frequency of surveillance could change from annual to an increased time interval between scans. This is supported by other research. A study to determine patients' perspectives of optimal intervals between scans using a patient decision aid found that participants with AAA sized 3.0–3.4 cm, and 4.5–4.9 cm, would choose to lengthen the current surveillance interval in the United Kingdom [17]. The key to being able to make these decisions was increasing patient knowledge and presenting patients with information on risk in an easy‐to‐understand format. Longer surveillance intervals for patients with small AAAs have been recommended by the European Society for Vascular Surgery [18].

In line with other research in this area [15, 19, 20], our study also identified that there were significant gaps in men's knowledge about AAA, the screening programme and AAA repair. This lack of knowledge and understanding has repercussions for the ability of men to make informed decisions about exiting surveillance, as they need to be knowledgeable about their condition to participate meaningfully in shared decision‐making. The benefits of shared decision‐making for patients are well established [21, 22] and have been recommended by UK AAA guidelines [23]. The use of a patient decision aid could be beneficial when helping men, and their families, make a decision about exiting surveillance.

### 4.1 Strengths and Limitations

There were two strengths of the research. First, as far as the authors are aware, no other countries have developed an exit strategy from AAA surveillance, so this study is novel. Second, we included a wide range of clinical disciplines in the workshop and a wide range of men and family members from areas with different levels of social deprivation in the interviews. It is noteworthy that 12 of the 22 men interviewed were from the two most socially deprived quintiles in the United Kingdom.

In terms of trustworthiness, we grounded the findings in the views of clinicians and men/family members and offer supporting quotes to enhance credibility. Our PPI Panel found the findings credible. We have described our research processes in detail to enhance dependability. Transferability of findings is likely to be good for the United Kingdom. The different health systems of other countries may limit transferability outside the United Kingdom. Transferability is likely to be poor for men from ethnic minority communities and limited for family members. There were no men from ethnic minority communities in the interview sample. Few men from ethnic minority communities are under surveillance in England because AAA is more prevalent in Caucasians. In addition, men had to have given permission to participate in research generally before we were able to contact them. It is possible that fewer men from ethic minority communities gave permission to participate in research generally. Further, we recruited fewer family members than planned, even after trying a different recruitment strategy using social media. It is unlikely that we reached data saturation after five interviews. Recruitment was challenging because the family members we approached for an interview often refused because they did not feel they knew about the man's AAA experience. It was interesting that a man and his family member could express different views about exiting surveillance. For example, three men supported the need for an exit strategy while their family members were opposed to it. Finally, confirmability was addressed within the research by having methodologists undertake the data collection and analysis. These methodologists did not have views about exiting AAA surveillance before starting the research. Clinical researchers on the team had views about exit, but were genuinely interested in the views of other clinicians and men/family members, so the findings are grounded in the data.

### 4.2 Implications

Neither clinicians nor men were happy with the current exit strategy in the NHS AAA screening programme in England. They identified a range of alternative strategies, including not exiting surveillance at all. The NHS screening programme could reconsider its current strategy, which is due to come into full use in 2028. Several alternatives to the current strategy, as well as their strengths and weaknesses, were identified. Making use of the existing Specialist Nurse appointment with men when they enter annual surveillance and then again when they enter 3‐monthly surveillance seems sensible for a discussion about potential exit. There may be a case for introducing a third nurse assessment when men's AAA reaches 5 cm to discuss their fitness for surgery in the future and how this might affect their decision about continuing with surveillance. Men may benefit from a decision aid similar to the one recently introduced to help men decide whether or not to have surgery for their AAA [24].

If the NHS AAA screening programme wishes to reconsider its exit strategy, it is important to understand men's preferences for different exit strategies. We had planned to undertake a telephone survey of men to measure their preferences, but we were concerned about men's low levels of knowledge about AAA, the screening programme and the existence of an exit strategy. We changed our plans based on findings from the study reported here and decided to design a Deliberative Engagement Session with men in AAA surveillance and their families, to identify preferences for exit [25]. We will report the results of this study in a future publication.

The findings of the study reported here could be potentially relevant and beneficial to other countries that provide AAA screening programmes, to men and women in local surveillance outside the NHS screening programme, and to patients undergoing surveillance for other conditions such as cancer.

### 4.3 Conclusions

Although clinicians and patients had conflicting views about the need for an exit strategy from AAA surveillance, they agreed that shared decision‐making was key to any exit strategy. The current exit strategy in the NHS AAA Screening Programme in England could be revisited to include options to exit if men feel they are at low risk of future rupture, and if men are unlikely to be healthy enough for future surgery. In addition, it could offer men the option of remaining under surveillance with reduced frequency of surveillance.

The views expressed are those of the authors and not necessarily those of the NIHR or the Department of Health and Social Care. For the purpose of open access, the author has applied a Creative Commons Attribution (CC BY) licence to any Author Accepted Manuscript version arising from this submission.

## Author Contributions

**Elizabeth Lumley:** conceptualisation, data curation, formal analysis, funding acquisition, investigation, methodology, project administration, resources, supervision, validation, visualisation, writing – original draft, review and editing. **Jane Hughes:** writing – review and editing. **Alan Elstone:** conceptualisation, funding acquisition, methodology, writing – review and editing. **Jo Hall:** conceptualisation, funding acquisition, methodology, writing – review and editing. **Niall MacGregor‐Smith:** conceptualisation, funding acquisition, methodology, writing – review and editing. **Jonathan Michaels:** conceptualisation, funding acquisition, methodology, writing – review and editing. **Akhtar Nasim:** conceptualisation, funding acquisition, methodology, writing – review and editing. **Stephen Radley:** conceptualisation, funding acquisition, methodology, writing – review and editing. **Phil Shackley:** conceptualisation, funding acquisition, methodology, writing – review and editing. **Gerry Stansby:** conceptualisation, funding acquisition, methodology, writing – review and editing. **Emily Wood:** conceptualisation, funding acquisition, investigation, methodology, writing – review and editing. **Alicia O'Cathain:** conceptualisation, data curation, formal analysis, funding acquisition, investigation, methodology, supervision, validation, visualisation, writing – review and editing.

## Disclosure

The views expressed are those of the authors and not necessarily those of the NIHR or the Department of Health and Social Care. For the purpose of open access, the author has applied a Creative Commons Attribution (CC BY) licence to any Author Accepted Manuscript version arising from this submission.

## Ethics Statement

Approval for the Clinical Stakeholder Workshop was received from the University of Sheffield Research Ethics Committee—application number 050427. Approval for the qualitative interviews with men in surveillance and their family members was granted by the WalesRec6 Ethics Committee 23/WA/0019, IRAS project ID 321528.

## Consent

All patients gave informed consent before their interview. They received full information, including a copy of the consent form, when initially approached about the study. Consent was taken in one of two ways: (1) in written format through completion and return of the consent form that they were sent, or (2) audio consent was recorded before the interview when the interviewer read out the questions from the consent form with the participant verbally indicating agreement with each statement then saying their name and the date at the end.

## Conflicts of Interest

In the interests of transparency, the authors would like to highlight that three of the authors have roles in the NHS AAA Screening Programme: Alan Elstone is the Professional Clinical Advisor (Nursing), Mr Akhtar Nasim is the National Surgical Lead and Professor Gerry Stansby is the Research Lead.

## Data Availability

The data that support the findings of this study are available upon request from the corresponding author. The data are not publicly available due to privacy or ethical restrictions.
